# Importance of support groups to the health and well-being of vulnerable children and young people living with HIV: a case study of the Kids Clubs program in Haiti

**DOI:** 10.1186/s12913-021-06242-5

**Published:** 2021-03-16

**Authors:** Susan K. Settergren, Robert Philippe, Joanne St. Louis, Nathaniel Segaren, Sylvie Boisson, Tessa Lewis, Olbeg Désinor, Kesner François

**Affiliations:** 1 Palladium, 1331 Pennsylvania Avenue NW, Suite 600, Washington, DC, 20004 USA; 2 Société d’Études et de Formation en Information Stratégique, Port-au-Prince, Haiti; 3 Caris Foundation, Port-au-Prince, Haiti; 4 United States Agency for International Development, Port-au-Prince, Haiti; 5 Ministère de la Santé Publique et de la Population, Port-au-Prince, Haiti

**Keywords:** Adolescents, ARV, Social support, Differentiated care, Viral suppression

## Abstract

**Background:**

Although access to antiretroviral therapy (ART) among children and young people living with HIV has increased in recent years, adherence to medication and viral suppression remain challenges. Evidence of benefits of support groups is growing and reflects a range of models and approaches. Since 2014, hospital-linked psychosocial support groups for children and young people living with HIV, known as Kids Clubs, have been established throughout Haiti. The program provides safe spaces for them to meet with peers, supports medication adherence, delivers health and life skills education, and facilitates linkages with clinic visits and social services. This study describes program enrollment and participant engagement, ART adherence and viral suppression among participants, and other outcomes attributed to the program by participants, caregivers, and program implementers.

**Methods:**

Our mixed methods study included quantitative analysis of program monitoring data on rollout and attendance, and medication adherence and viral load results extracted from medical records. We collected qualitative data from club members, caregivers, and implementers about their experiences with the clubs and the impact of participation.

**Results:**

From January 2014–December 2018, 1330 individuals aged 8–29 were enrolled in the program; over three-quarters participated for at least 12 months. In 2018, 1038 members attended at least one club meeting; more than half missed three or fewer monthly meetings. Three-quarters of ever-enrolled members reported consistent medication use at their most recent clinic visit; 64.2% (600/935) of those with a recent viral load test were virally suppressed. Level of club attendance was positively associated with ART adherence (*p* < 0.01) and viral suppression (*p* < 0.05). Club members, caregivers, and implementers noted the value of the clubs to participants’ retention in care and medication adherence, health knowledge, and capacity to deal with peer pressure, stigma, shyness, and depression.

**Conclusions:**

The Kids Club program has been successful in scaling HIV support services to highly vulnerable children and young people through peer-based groups, and program participation has led to a range of benefits. Efforts to innovate, evaluate, and scale support strategies for vulnerable young populations must be accelerated in order to ensure that they survive, thrive, and reach their full potential.

## Background

While children and young people’s access to antiretroviral therapy (ART) has increased dramatically over the past decade, ART adherence and viral suppression remain challenges, especially among highly vulnerable populations in lower- and middle-income countries [1–6]. Nondisclosure and lack of routine have been identified as contributing to poor medication adherence and retention in care. These factors are compounded by cognitive, emotional, and social changes associated with the transition from childhood to adulthood, as well as stigma surrounding HIV, including within the school environment [7–14]. Support groups have emerged as a feasible strategy, and evidence of their impact on the health and well-being of children, adolescents, and youth is growing [15–20]. Several approaches have been explored, including those singularly focused on promoting ART adherence [21, 22], as well as inclusion of a group intervention as part of a range of broader child- and youth-friendly, comprehensive, differentiated care packages of services [23–27].

Haiti had an estimated 7800 10–19-year-olds living with HIV in 2019, 45% of whom were receiving ART [1]. Promotion of psychosocial care and integration of people living with HIV and affected families into social and public assistance programs supported by the government and partners is a national priority [28]. Since 2014, Caris Foundation, a nonprofit private foundation, has provided hospital-linked psychosocial support to children and young people on ART through two projects, Bien Et ak Santé Timoun (2014–2018) and Project Impact Youth (2019–2023), both funded by the United States President’s Emergency Plan for AIDS Relief through the United States Agency for International Development. Through its Kid Club program, which comprises peer-based clubs tailored to three age groups, it aims to help children and young people stay in contact with health services. The clubs meet monthly and also serve as a conduit for delivery of other services, including health and life skills education and hygiene kits. Trained nurses implement the clubs using a standardized curriculum that mixes interactive learning with recreational activities. A hot meal and transport fees are provided. As of December 2019, 54 Kids Clubs had been established at 24 hospital locations throughout the country [29]. We conducted a retrospective case study of the program to better understand program rollout, participants’ engagement with the clubs, and the program’s contributions to members’ ART adherence, viral suppression, and other well-being outcomes.

## Methods

### Study design

We used a mixed methods design for the case study. Our outcomes of interest were club enrollment and attendance, ART adherence and viral suppression among club members, and other club contributions to the health and well-being of participants. We conducted quantitative secondary analysis of Caris Foundation project monitoring data and linked medical record information for all individuals ever enrolled in the 54 clubs during 2014–2018. We purposively sampled 10 clubs at five locations for in-depth qualitative data collection based on geographic diversity, accessibility, size, and types of clubs offered (Table 1). At each location, we conducted focus group discussions (FGDs) separately with club members, and their caregivers, and key informant interviews with nurse coordinators implementing the program.

**Table 1:** Sites selected for in-depth study

Site	Department	Club types	Number of club members who attended at least one club meeting in 2018
Grace Children’s Hospital	Ouest	13- to 17-year-olds	23
Hôpital Bernard Mevs	Ouest	9- to 12-year-olds	14
Hôpital Saint Damien Nos Petits Frères et Soeurs^a^	Ouest	11-year-olds	112
		15- to 17-year-olds	71
Hôpital Immaculée Conception des Cayes	Sud	9- to 12-year-olds	76
		13- to 17-year-olds	30
		18-year-olds +	26
Hôpital Sacré Coeur de Milot	Nord	9- to 12-year-olds	20
		13- to 17-year-olds	20
		18-year-olds +	11

^a^Due to the large number of enrolled children, clubs at this site are defined by narrower age groups than the three types implemented at other sites

### Data collection

Quantitative data on all individuals enrolled in the program January 2014–December 2018 were obtained from project monitoring databases. Variables included socio-demographic and historical club attendance information, all of which were complete for individuals who were ever enrolled in a club during the time period. Clinical information for these individuals was extracted from the Ministère de la Santé Publique et de la Population (MSPP) iSanté national electronic medical record central server database. Data were abstracted from client registration, intake, ART adherence, and laboratory forms for the most recent clinic visit during the three-year period prior to the time of data extraction (i.e., 1 September 2016–31 August 2019). Intake forms were available for 77.1% (1026/1330) of Kids Club enrollees.

For each of the 10 clubs selected for in-depth qualitative study, we randomly selected 12 club members who were highly active during January–December 2018—defined as having missed three or fewer club meetings—and invited them to participate in a FGD about their experiences with the program. Caregivers of these individuals (with the exception of those in the two 18-year-olds + clubs) were also invited to participate in separate FGDs. Invitations were extended a week before the scheduled FGDs. FGD topics included club activities, attendance, challenges to participation, membership benefits, and feelings about the club. FGD guides for club members were tailored to the age group of the club and elicited individual member’s perspectives. Caregivers of club members were asked about their observations and perspectives regarding members’ club participation. In total, 96 children and young people (54 female, 42 male) participated in the 10 club member FGDs, which ranged in size from seven to 12 individuals. A total of 69 caregivers (mostly female) participated in the eight caregiver FGDs, which ranged in size from seven to 10 individuals. Many caregivers were not the biological parents of the club members, but rather other family members or legal guardians. The 10 nurse coordinators who implemented the selected clubs (all female) were interviewed individually or in groups of two to four, depending on how the site was staffed. Semi-structured interview guides covered the same topics as the FGDs, focusing on implementer observations and perspectives regarding member participation. All tools were developed in English and then translated, piloted, and administered in Creole. Eight data collectors with prior child and adolescent qualitative research experience were trained by the study team and collected all qualitative data. Working in pairs, one person led the FGD or interview, while the other took summary notes and audio recorded the session. All qualitative data collection took place 04 June–26 July 2019 at the club meeting locations and each session ranged in length from 60 to 90 min.

### Data analysis

Project monitoring and clinical data were analyzed using IBM SPSS Statistics version 25. Consistency and range checks were performed separately on each data source, and key fields (date of birth, sex, and club meeting/clinic visit dates) were cross-checked during merging of Kids Club and clinic records. Unless otherwise noted in the findings, all available data were used in analysis of the clinical data. Chi-square and Fisher’s Exact tests were used to test differences among categorical variables.

Data collectors transcribed the FGD and interview recordings in Creole and translated them into English. They and the lead author coded these data by analysis topic using Atlas.ti 8. Using an inductive approach, they summarized themes within topics by data source and then concatenated the data from all sources by common theme. Quotations supporting the themes were extracted from the transcripts and identified by club and type of FGD participant (i.e., club member or caregiver). To maintain anonymity of the nurse coordinators, codes were randomly assigned when reporting attribution.

### Ethical review

Ethics review was conducted, and approval granted by the Haiti Comité National de Bioéthique, MSPP, and the U.S.-based Health Media Lab. Written informed consent was obtained from all participants ages 18 and older. For participants under 18 years, written informed consent was sought from the caregiver and assent was obtained from the adolescent.

## Results

### Club enrollment

Across the entire program, secondary analysis of project monitoring data showed that 1130 individuals had been enrolled in the Kids Club from 2014 to 2018, with nearly half (43.8%) located in the Ouest department (Table 2). The rate of enrollment was highest during 2015–2017, when about three-quarters of club members joined a club. Most club members (81.0%) had been seen at an HIV clinic before the start of the program in 2014. Females comprised a slightly larger percentage (52.9%) of those ever enrolled. About one-third of enrollees (35.5%) began attending a club at ages 8–12, while 43.5% were ages 13–17 and 21.0% were ages 18–29. As of December 2018, 43.2% last attended a 9- to 12-year-old club, while 42.3 and 14.4% last attended 13- to 17-year-old and 18-year-old + clubs, respectively. Characteristics among the subpopulation with available clinical data were similar to those of the ever-enrolled group.

**Table 2:** Characteristics of Kids Club participants

Club participant characteristics	Ever enrolled (***N*** = 1330) % (n)	HIV patient intake form available (***N*** = 1026) % (n)
**Department**		
Artibonite	2.6 (34)	0.6 (6)
Centre	3.0 (40)	3.7 (38)
Grand’Anse	4.4 (58)	5.3 (54)
Nippes	3.7 (49)	4.8 (49)
Nord	15.3 (203)	18.1 (186)
Nord-Est	1.8 (24)	1.3 (13)
Nord-Ouest	7.9 (105)	6.3 (65)
Ouest	43.7 (581)	39.5 (405)
Sud	14.4 (191)	16.3 (167)
Sud-Est	3.4 (45)	4.2 (43)
**Year of club enrollment (Jan–Dec)**		
2014	11.1 (148)	11.1 (114)
2015	31.7 (422)	31.5 (323)
2016	25.3 (336)	28.2 (289)
2017	20.5 (273)	19.3 (198)
2018	11.4 (151)	9.9 (102)
**Year of initial visit at a health facility**		
Before 2014	–	81.0 (831)
2014	–	5.5 (56)
2015	–	6.3 (65)
2016	–	4.5 (46)
2017	–	2.1 (22)
2018	–	0.5 (6)
**Sex**		
Female	52.9 (703)	51.9 (542)
Male	47.1 (627)	48.1 (503)
**Age at club enrollment**^**a**^		
8–12 years	35.5 (472)	37.9 (389)
13–17 years	43.5 (579)	42.5 (436)
18–29 years	21.0 (279)	19.6 (201)
**Last club type attended**		
9- to 12-year-old	43.2 (575)	45.9 (471)
13- to 17-year-old	42.3 (563)	40.7 (418)
18-year-old +	14.4 (192)	13.4 (137)

Source: Caris Foundation project monitoring database and clinic records

^a^Calculated based on date of birth and date of first club meeting

### Club attendance

About three-quarters of those ever enrolled in a club (77.6%) participated (or were exposed to the club) for at least one year, attending a minimum of two club meetings and with the time between the first and last attendance dates spanning at least 12 months (Table 3). This one-year minimum participation rate varied by department, year of enrollment, participant’s age, and club type (*p* < 0.001), but not by sex. Only those in the 2018 cohort who were enrolled in January could qualify for at least 12 months of participation; thus, as expected, this cohort had the lowest minimum participation rate.

**Table 3:** Club attendance

Club participant characteristics^**a**^	Participated for at least 12 months^**b**^ % (n)	Active in 2018^**c**^ % (n)	Highly active in 2018^**d**^ % (n)
**Department**			
Artibonite	52.9 (18/34)***	100.0 (34/34)***	90.0 (18/20)***
Centre	80.0 (32/40)	100.0 (40/40)	87.5 (28/32)
Grand’Anse	84.5 (49/58)	77.6 (45/58)	58.5 (31/53)
Nippes	46.9 (23/49)	69.4 (34/49)	44.7 (17/38)
Nord	67.5 (137/203)	57.6 (117/203)	41.8 (79/189)
Nord-Est	70.8 (17/24)	100.0 (24/24)	83.3 (10/12)
Nord-Ouest	81.9 (86/105)	76.2 (80/105)	58.0 (51/88)
Ouest	81.6 (474/581)	79.5 (462/581)	50.6 (269/532)
Sud	84.3 (161/191)	86.9 (166/191)	65.5 (112/171)
Sud-Est	77.8 (35/45)	80.0 (36/45)	70.5 (31/44)
**Year of club enrollment**			
2014	99.3 (147/148)***	78.4 (116/148)***	65.5 (97/148)***
2015	94.8 (400/422)	69.4 (293/422)	47.9 (202/422)
2016	70.2 (236/336)	66.7 (224/336)	44.9 (151/336)
2017	87.2 (238/273)	93.0 (254/273)	71.8 (196/273)
2018	7.3 (11/151)^e^	100.0 (151/151)	–
**Sex**			
Female	76.7 (539/703)	77.0 (541/703)	53.3 (334/627)
Male	78.6 (493/703)	79.3 (497/627)	56.5 (312/552)
**Age at club enrollment**			
8–12 years	68.9 (321/472)***	81.8 (386/472)***	61.4 (235/383)***
13–17 years	83.8 (485/579)	80.8 (468/579)	57.8 (307/531)
18–29 years	81.0 (226/279)	65.9 (184/279)	39.2 (104/265)
**Last club type attended**			
9- to 12-year-old	70.6 (406/575)***	83.1 (478/575)***	62.8 (299/476)***
13- to 17-year-old	81.7 (460/563)	76.2 (429/563)	52.6 (275/523)
18-year-old +	86.5 (166/192)	68.2 (131/192)	40.0 (72/180)
**Total**	**77.6 (1032/1330)**	**78.0 (1038/1330)**	**54.8 (646/1179)**

Source: Project monitoring database

***Differences among the groups are statistically significant at *p* < 0.001

^a^All club enrollees were included in the attendance analyses unless otherwise noted, including those whose participation ended at any point due to severe illness or death

^b^Difference between last club meeting date and first club meeting date ≥12 months

^c^Attended at least one club meeting in 2018

^d^Among those enrolled through December 2017, did not miss more than three club meetings from January–December 2018

^e^Includes only those enrolled in January 2018

Seventy-eight percent (1038/1330) of individuals ever enrolled in a club were active during the 2018 calendar year by attending at least one club meeting that year; 2018 attendance varied by department, year of enrollment, participant’s age, and club type (*p* < 0.001), but not by sex. The project defined highly active club members as those who had not missed more than three club visits within a 12-month time period. Highly active participation in 2018 was examined among the subset of 1179 children and young people enrolled in the Kids Club program as of December 2017. Slightly more than half (54.8%) had participated actively in 2018. Highly active participation varied by all measured characteristics except sex, following patterns similar to those for active participation (i.e., measured by at least one visit in 2018). Members enrolled throughout the five-year project period attended 16.2 club meetings on average, with a range of 1 to 51 meetings.

Club attendance was reported to be a priority by the club members participating in the FGDs. They shared that they rarely missed a club meeting and were unhappy if unable to attend, because they missed their friends and the topics discussed (Table 4). They also expressed a desire to continue to attend the clubs for the indefinite future. Caregivers, too, emphasized their commitment to club attendance. There was broad agreement among nurse coordinators, caregivers, and club members that street violence, political unrest, lack of resources (primarily for transportation), school schedule conflicts (especially during exam periods), and heavy road traffic contributed to members missing club meetings. Other reasons included summer holidays and illness. Scheduling conflicts with employment and college attendance were reported to contribute to irregular attendance among some youth in the 18-year-old + clubs. Nurse coordinators noted pregnancy and subsequent geographic relocation as reasons for adolescent girls dropping out of clubs.

**Table 4:** Quotes on Kids Club attendance

**Club attendance is a priority**	
*“Because I’m attached to the club, I come every time.”* (Member of 9- to 12-year-old club, Hôpital Immaculée Conception des Cayes)	
*“I feel comfortable in the club, I have a lot of good times, I want to stay for as long as it exists.”* (Member of 18-year-old + club, Hôpital Sacré Coeur de Milot)	
*“When the time comes, steal or borrow, the child must come to the club* [laughter]*. Even if you are doing the laundry, you have to leave it and go to the club.”* (Caregiver of member of 9- to 12-year-old club, Hôpital Sacré Coeur de Milot)	
**Reasons for missing club meetings or dropping out**	
*“The difficulties are when … I hear shots and people run from one side to the other. At this moment, it is difficult to find a bus to reach the meeting on time.”* (Member of 9- to 12-year-old club, Hôpital Immaculée Conception des Cayes)	
*“The difficulty that we find is the transportation fee, because there are some of us who don’t have a mother or father; if we don’t have money to pay a vehicle or motorcycle, it’s difficult to come to the club.”* (Member of 18-year-old + club, Hôpital Sacré Coeur de Milot)	
*“The only problem* [with attendance] *is when you go to pick up the child when he is attending a school course, when he is in examination period … This may cause him to miss a day and not come. But if there is no school, there is no problem.”* (Member of 9- to 12-year-old club, Hôpital Immaculée Conception des Cayes)	
*“Sometimes I am sick* [and can’t come to a club meeting]*.”* (Member of 9- to 12-year-old club, Hôpital Saint Damien Nos Petits Freres et Soeurs)	
*“The children who miss the meetings more are some in the 18-year-old + groups because some of them work, and some of them have finished secondary school and go to college.”* (Nurse coordinator, Group D)	
*“A reason that causes children* [to stop attending the club meeting]*, for the girls, is they get pregnant. Sometimes they are moved to live farther away or their parents leave the department.”* (Nurse coordinator, Group A)	

### ART adherence

Recent clinical data on self-reported ART adherence (i.e., for visits occurring 01 September 2016–31 August 2019) were available for 62% (833/1330) of all individuals ever enrolled in a Kids Club. Among them, 77.2% reported taking 100% of their doses in the month before their last visit, and 73.1% reported no missed doses in the preceding 4 days, both of which were questions asked during routine clinic visits (Table 5). Both adherence measures were found to differ by department of the club location (*p* < 0.001), although in some cases sample sizes were small and estimates should be interpreted with caution. Adherence also differed by club enrollment cohort with the lowest percentage of adherers for both measures among the 2015 cohort (*p* < 0.05). Differences by sex, and among ages and club types, were small and not statistically significant. No differences in adherence were found by length of program participation, but both adherence measures were associated with number of club meetings attended—84.0% of those who attended at least 24 club meetings reported 100% doses taken in the past month, and 78.6% reported no missed doses in the past 4 days, compared to 75.1 and 71.3% (*p* < 0.01 and *p* < 0.05, respectively) among those attending fewer meetings. Active participation in 2018 was also associated with the two adherence measures—80.1% of active members reported 100% adherence, and 75.0% reported no missed doses, compared to 67.0% and 66.1% (*p* < 0.001 and *p* < 0.01, respectively) among those inactive in 2018. Similar differences were found for those highly active in 2018.

**Table 5:** ART adherence by club participant characteristics

Club participant characteristics	Reported 100% doses taken in past month^**a**^	Reported no missed doses in past 4 days^**a**^	Virally suppressed^**cb**^
	% (n/N)	% (n/N)	% (n/N)
**Department**			
Artibonite	20.0 (1/5)***	50.0 (1/2)***	16.7 (1/6)***
Centre	97.4 (38/39)	97.4 (38/39)	40.5 (15/37)
Grand’Anse	98.1 (52/53)	100.0 (53/53)	55.6 (25/45)
Nippes	74.4 (32/43)	80.6 (29/36)	53.5 (23/43)
Nord	63.4 (111/175)	60.2 (106/176)	50.6 (86/170)
Nord-Est	92.3 (12/13)	92.3 (12/13)	55.6 (5/9)
Nord-Ouest	82.1 (55/67)	91.1 (41/45)	59.7 (37/62)
Ouest	74.0 (214/289)	67.6 (198/293)	75.6 (279/369)
Sud	88.1 (89/101)	74.1 (100/135)	69.0 (107/155)
Sud-Est	83.9 (26/31)	75.6 (31/41)	56.4 (22/39)
**Year of club enrollment**			
2014	85.2* (69/81)	78.9* (75/95)	67.7 (67/99)
2015	71.6 (169/236)	66.0 (159/241)	67.6 (196/290)
2016	74.4 (180/242)	73.3 (178/243)	58.9 (156/265)
2017	82.3 (135/164)	74.9 (131/175)	62.9 (117/186)
2018	82.8 (77/93)	83.4 (66/79)	67.4 (64/95)
**Sex**			
Female	75.7 (321/424)	72.7 (312/429)	64.6 (294/455)
Male	78.8 (309/392)	73.5 (297/404)	64.8 (306/480)
**Age at club enrollment**			
8–12 years	78.0 (234/300)	71.7 (226/315)	65.5 (235/359)
13–17 years	74.4 (256/344)	72.2 (252/349)	63.1 (251/398)
18–29 years	81.4 (140/172)	77.5 (131/169)	64.0 (114/178)
**Club type at last visit**			
9- to 12-year-old	78.9 (284/360)	73.0 (276/378)	66.7 (291/436)
13- to 17-year-old	73.7 (255/346)	72.3 (248/343)	60.2 (228/379)
18-year-old +	82.7 (91/110)	75.9 (85/112)	67.5 (81/120)
**Length of club participation**			
< 12 months	73.8 (141/191)	75.6 (133/176)	63.1 (130/206)
12–24 months	78.4 (171/218)	73.2 (167/228)	60.5 (135/223)
24+ months	78.1 (318/407)	72.0 (309/429)	66.2 (335/506)
**Total number of club meetings attended**			
< 24	75.1 (467/622)**	71.3 (444/623)*	62.4 (424/680)*
24+	84.0 (163/194)	78.6 (165/210)	69.0 (176/255)
**Active in a club in 2018**^**c**^			
Yes	80.1 (510/637)***	75.0 (490/653)**	65.8 (498/757)*
No	67.0 (120/179)	66.1 (119/180)	57.3 (102/178)
**Highly active in a club in 2018**^**d**^			
Yes	80.5 (327/406)**	74.8 (324/433)*	65.5 (325/496)
No	71.3 (226/317)	68.2 (219/321)	61.3 (211/344)
**Total**	**77.2 (630/816)**	**73.1 (609/833)**	**64.2 (600/935)**

*, **, *** Differences among the groups are statistically significant at *p* < 0.05, *p* < 0.01, and *p* < 0.001, respectively

^a^At most recent clinical visit within the three-year period (01 September 2016–31 August 2019)

^b^Most recent viral load test within the three-year period (01 September 2016–31 August 2019); 94.8% of the most recent tests occurred in 2018 and 2019

^c^Attended at least one club meeting in 2018

^d^Among those enrolled before January 2018, did not miss more than three club meetings from January–December 2018

Club member FGD participants, who were highly active in 2018, noted the value of medication adherence messaging at the club meetings (Table 6). Older members referred to benefits of adherence support through peer networks within the clubs. Caregivers also described how participation in the Kids Club had led to improvements among those under their care taking their medication.

**Table 6:** Quotes on Kids Club contribution to medication adherence

Improved medication adherence	
*“When they treat the topic* [of taking medication] *with me, I feel well.” (Member of* 9- to 12-year-old club, Hôpital Sacré Coeur de Milot)	
*“Before the topics they discussed with us regarding the medication, I used to not take them, but now I take them, it changed me a lot.”* (Member of 13- to 17-year-old club, Hôpital Sacré Coeur de Milot)	
*“When my grandmother gave me the medicines, I threw them away, I didn’t drink them. When I came here, the nurses talked to me; after that, I saw how the club works, and I followed the information, I began to improve and drink the medications.* (Member of 18-year-old + club, Hôpital Sacré Coeur de Milot)	
*“When we get into the club, we share that* [we are living with HIV], *and when we notice some teens who don’t feel well, sometimes they are too weak, we can talk to them and when they explain to us, we can encourage them to take medicine and talk to them.”* (Member of 18-year-old + club, Hôpital Immaculée Conception des Cayes)	
*“The club helps to manage the health of children; sometimes children are repugnant to drugs, but when they participate in the club, they even encourage us to give them the drugs because the nurses tell them to take them every day.” (*Caregiver of member of 9- to 12-year-old club, Hôpital Bernard Mevs)	

### Viral suppression

Approximately 70% of 1130 individuals ever enrolled in a Kids Club had at least one viral load test performed 01 September 2016–31 August 2019, with results available on the iSanté central server (Table 5). Among them, 64.2% were virally suppressed (i.e., had a viral load result of less than 1000 copies/ml) at the time of their most recent test. Viral load suppression varied considerably by geographic location, ranging from 16.7% in Artibonite to 75.6% in Ouest, *p* < 0.001, although small sample sizes for some departments warrant caution in interpretation. Differences in viral load suppression among enrollment cohorts, age groups, and club types, and by sex, were small and not statistically significant. Although a larger percentage of those who had participated in the program for at least 24 months were virally suppressed compared to those who participated for shorter periods, the differences were not statistically significant. However, those who attended at least 24 club meetings were more likely to be virally suppressed than those who attended fewer meetings (69.0% vs. 62.4%, respectively, *p* < 0.05), as were active members in 2018 compared to those inactive (65.8 and 57.3%, respectively, *p* < 0.05). A similar difference was found for those highly active compared to those not highly active, but the difference was not statistically significant. Viral load suppression was highly associated with the two self-reported adherence measures (Table 7).

**Table 7:** Association between self-reported adherence and viral load suppression

Adherence measures^a^	Virally suppressed^**b**^ % (n/N)
**Reported 100% doses taken in past month**	
Yes	67.7 (401/592)***
No	43.0 (77/179)
**Reported no missed doses in past 4 days**	
Yes	66.8 (380/569)***
No	53.2 (215/216)

*** Differences between the groups are statistically significant at *p* < 0.001

^a^At most recent clinical visit within the three-year period (01 September 2016–31 August 2019)

^b^Most recent viral load test within the three-year period (01 September 2016–31 August 2019)

### Other club contributions

Most club members and caregivers discussed the importance and value of the education component of the club meetings and described a range of topics taught and the knowledge they had gained (Table 8). HIV, medication adherence, hygiene, and sex education and contraception were among the most commonly cited and appreciated health related topics. Club members, caregivers, and implementers also emphasized the value of educational sessions aimed at promoting psychosocial development including dealing with peer pressure, respect for others, self-esteem, and human rights. Club members of all ages reported gaining a great deal of knowledge through their participation in the clubs. Older participants were more likely than younger ones to say that this learning had changed their lives for the better. However, caregivers often described positive behavioural changes among younger children as well.

**Table 8:** Quotes on the value of the Kids Club education component

**Health education**	
*“The club taught us how we got the disease and what measures to take.”* (Member of 13- to 17-year-old club, Grace Children’s Hospital)	
*“They say how to take care of our bodies, especially when you have menstruation; not to have sex.”* (Member of 9- to 12-year-old club, Hôpital Sacré Coeur de Milot)	
*They talk about different types of contraceptive methods. They even taught us abstinence* [smiling]. *Also, they told us that we might not be able to wait; they gave us different methods to use.”* (Member of 13- to 17-year-old club, Hôpital Immaculée Conception des Cayes)	
**Psychosocial development**	
*“They taught us how to value ourselves … like some children behave as if they don’t exist. They stay in* [the] *corner. But they taught us how to stand up for ourselves, to be proud of ourselves. So that we know we are human, that we are living.”* (Member of 13- to 17-year-old club, Hôpital Immaculée Conception des Cayes)	
*“They taught us how to respect ourselves, how to respect other people. We must respect our parents. How to respect other people’s opinion.”* (Member of 13- to 17-year-old club, Hôpital Immaculée Conception des Cayes)	
*“When children come to the club, they receive good training. They learn how to talk to their friends, neighbors,* [and parents] *… When the neighbors say that you have a good child who has good behaviour … when they congratulate your child, it is very important to us.”* (Caregiver of child in 9- to 12-year-old club, Hôpital Immaculée Conception des Cayes)	
*“Last Saturday, the topic was on how to behave in society, how to claim our rights.”* (Member of 18-year-old + club, Hôpital Immaculée Conception des Cayes)	
**Learning and behaviour change**	
*“What I learn is in relation to the subjects that they teach. Now, when I put them into practice, I have different behavior from the way I was before. It is in relation to the subjects* [they teach] *that my life has changed.”* (Member of 18-year-old + club, Hôpital Immaculée Conception des Cayes)	
*“Before, I was really careless, I did not take anything seriously except school. When I started to understand and think about everything I learned in the club, I realized that I must take everything seriously.”* (Member of 13- to 17-year-old club, Grace Children’s Hospital)	
*“There are things that they talked about, like alcohol. I used to have friends who were forcing me to drink alcohol, I took my distance from them, I do it less now and I have changed.”* (Member of 13- to 17-year-old club, Hôpital Sacré Coeur de Milot)	

Club members and caregivers spoke of the contribution of the Kids Clubs to club members’ psychosocial development and well-being through group interaction as well as the education sessions to overcome shyness and improve self-esteem, offer encouragement and hope, and instill happiness in their lives (Table 9). Those who were aware they are living with HIV talked about how their participation in a club helped them better understand the disease, how to stay healthy, and how to live positively.

**Table 9:** Quotes on Kids Club contributions to psychosocial development

**Shyness and self-esteem**	
*I have a child who was very shy and I always encouraged her to attend the club, where there are some club activities. I told her it would help her to get developed instead of staying home. She was afraid and she told me there are many children and they would look down on her. I told her, ‘No, they are all like you,’ and she did not have to be afraid. It encouraged her and thanks to that, she is no longer timid.”* (Caregiver of member of 13- to 17-year-old club, Hôpital Sacré Coeur de Milot)	
*“They show them how to live, they tell them that they have the same value as everyone; they don’t need to … worry because of the disease.”* (Caregiver of member of 9- to 12-year-old club, Hôpital Sacré Coeur de Milot)	
*“There was a way the child did not develop before. But they give them training, take them to other areas; they meet little friends … see new images*... [that’s] *what develops their minds.”* (Caregiver of member of 9- to 12-year-old club, Hôpital Bernard Mevs)	
**Encouragement and hope**	
*“It helps them, it gives them the desire to live, they are not discouraged when they see that others care about them. It encourages them, they are not abandoned … It means that the child is not a lost cause.”* (Caregiver of member of 9- to 12-year-old club, Hôpital Immaculée Conception des Cayes)	
*“She tells me that she wants to become a nurse. But there was someone who told her that [because of] the illness she could not become a nurse … and that she couldn’t go on any trip because of the illness. When she comes to the club, they say it is not true; there is nothing you cannot do because [of] your illness.” (Caregiver of member of 13- to 17-year-old club, Hôpital Immaculée Conception des Cayes)*	
*The advice they give us through the subjects about this disease, not just telling us about its treatment, but the way they describe it, shows us how to behave facing this disease. I found it very important because when one is living with this kind of sickness it may lead to suicide. This* [advice] *helps me personally to move on, it also allows me to believe in my future, to believe that I can do a lot of things, and that the disease that I have cannot stop me.* (Youth in 18-year-old + club, Hôpital Immaculée Conception des Cayes)	
**Happiness and well-being**	
*The children are happy, they are no longer in sadness.* (Caregiver of member of 9- to 12-year-old club, Hôpital Bernard Mevs)	
*“They taught us how to live comfortably in society, how to be a responsible man, and then to live well, better.”* (Member of 13- to 17-year-old club, Grace Children’s Hospital)	

## Discussion

This study provided an in-depth look into the participation of highly vulnerable children and young people in a large-scale, peer-based support group intervention implemented as a component of a comprehensive package of medical, behavioral, and structural interventions. In this regard, it contributes to the growing body of evidence on differentiated care models that aim to holistically address the multitude of challenges children and young people living with HIV face.

The Kids Club program demonstrated the feasibility of scaling support groups, while maintaining high levels of participation. The number of health facilities hosting clubs grew from six to 24, and enrollment increased nearly tenfold over the five-year period with clubs established in all 10 administrative departments. In 2018, 1038 children and young people had attended at least one club meeting, representing one-third of the estimated number of comparably aged individuals in Haiti on ART—with highly active engagement of roughly 20% of this population.

The clubs included members as young as 8 years and as old as 29 years, with the majority (79%) under the age of 18. Roughly equal numbers of female and male adolescents participated. Age and sex composition was comparable to those targeted by other recently studied support interventions for young people, although some were more narrowly focused [20]. FANMI, a combination program approach implemented at Haiti’s largest HIV treatment clinic and piloted among ages 10–20 reported higher female than male participation (i.e., 76% female) and a median participant age of 17.5 years [27]. A study currently is underway testing the efficacy of FANMI among adolescent girls and young women ages 16–23 [30]. Most Kids Club members (81%) had initiated HIV care at a health facility at least one year before the start of the Kids Club program in 2014. Initiation of clinical monitoring among Kids Club members at a young age (i.e., more than one-third of club members were enrolled at age 12 or younger) reflects the high proportion of club members who had acquired HIV through vertical transmission.

Once enrolled, both female and male members tended to stay in the clubs. More than three-quarters of those enrolled over the five-year period participated for at least 12 months and attended an average of 16 club meetings. Those in the 9- to 12-year-old club were least likely to have participated for 12 months but also more likely to be active in 2018 compared to members in the older age clubs. Closer analysis showed that some club type differences could be explained by the fact that members of the youngest age group made up more than half of the 2018 cohort and thus did not have the opportunity to participate for at least 12 months. While the majority of members of the 18-year-old + club (86%) remained in a club for at least a year, only about 40% were actively participating in 2018, suggesting that older members may face additional challenges to routine club participation, an issue raised by FGD participants related to conflicts with employment schedules, pressures to find work, and college attendance. Limitations in abstracting individual medical records prevented our analysis of retention in clinical care. However, given that nurse coordinators link closely with clinics to ensure participants keep their appointments, club attendance could be considered a proxy for retention in care. One-year club attendance of 78% compares favourably with the 66% one-year post-ART retention rate observed among individuals receiving adolescent friendly services at Haiti’s largest HIV clinic [26].

Reports from club members regarding the personal importance of the club and the desire to attend meetings substantiated attendance records. In spite of facing considerable obstacles in getting to a club meeting, they and their caregivers expressed strong commitment to participate. Attendance among all age groups was driven by the desire to learn about the educational topics delivered, participate in the recreational events hosted, and benefit from the supportive atmosphere of the clubs. FGD participants reported learning about puberty, personal hygiene, and HIV (reportedly popular topics), and how to handle peer pressure and stigma and overcome shyness. Many examples cited by club members, their caregivers, and program staff indicated the importance of this learning in club participants’ lives and the behavior change it motivated. The value of the professed joy the clubs brought to the lives of club participants also cannot be underestimated. Participants said that they “felt better” as the result of being in the Kids Club. This improved sense of well-being likely resulted from many factors, including fewer struggles at home over taking medications, opportunities to socialize with peers in a stigma-free environment, and better knowledge and understanding of, and skills to, positively face the physical, psychological, and social ups and downs of adolescence. These findings lend support to previous studies that have also demonstrated psychosocial benefits of adolescent HIV support interventions [23].

About three-quarters of ever-enrolled club members self-reported high levels of ART adherence at their most recent clinic visit, similar to a community-based adolescent ART support intervention in Zimbabwe [23]. Self-reported adherence was found to be associated with Kids Club participation, measured in total number of meetings attended and participation in 2018, suggesting a possible dose-response effect. Among Kids Club members with a recent viral load test, 64% were virally suppressed—a proportion somewhat higher than that reported in the FANMI study (i.e., 55% post intervention) [27] and a study at six Haitian hospitals that reported 50% virologic suppression among 13–21 year-olds [31]. In addition, a recent national cohort analysis of those initiated on ART in 2016 and 2017 found 45% virologic suppression among 10–14-year-olds in both cohorts and 50 and 87% among 15–19 year-olds in the 2016 and 2017 cohorts, respectively [32]. In our study, we found no differences between age groups or female and male club members. Similar to ART adherence findings, viral suppression was associated with number of club meetings attended and club attendance in 2018. Viral suppression was found to be positively associated with ART adherence, suggesting that at least some of the association of club attendance and viral suppression was mediated through ART adherence.

Our study had several limitations. Given the absence of a counterfactual, observed benefits cannot be directly attributed to the program and the direction or source of influence of the association between club attendance and adherence and viral suppression cannot be determined conclusively. Club members adherent to their medication may have been more likely to actively participate in the program than nonadherent children because they felt better. External factors, such as family care or access to the clinic and club location, may have mediated both club participation and adherence. Additionally, clinical data were available for only three-quarters of Kids Club members. Although the subset with clinical data did not appear to differ greatly from the full sample on characteristics that could be assessed, the influence of other factors and the extent of bias they may have introduced in the study is unknown. Furthermore, qualitative, in-depth analysis was based on a limited number of clubs. Although responses were consistently similar across the sites sampled, they may not be representative of all sites. Geographic variation in club participation, as well as ART adherence and viral suppression, was observed, warranting further study.

## Conclusions

Our study contributes to the growing evidence of the importance of psychosocial support as a component of comprehensive programming for children and young people living with HIV in order to support ART adherence and virologic suppression. As advocated by others, further study of the psychosocial components of combination care models, such as the Kids Club program, is needed to understand the pathways through which they influence health and well-being and how those pathways can be shaped to help young populations living with HIV survive, thrive, and reach their full potential.

## Data Availability

The datasets used and/or analysed during the current study are available from the corresponding author on reasonable request.

## Abbreviations

ART: Antiretroviral therapy
FGD: Focus group discussion
MSPP: Ministère de la Santé Publique et de la Population
